# Adsorption Characteristics of Pristine and Magnetic Olive Stones Biochar with Respect to Clofazimine

**DOI:** 10.3390/nano11040963

**Published:** 2021-04-09

**Authors:** Marwa El-Azazy, Iman Nabil, Siham S. Hassan, Ahmed S. El-Shafie

**Affiliations:** Department of Chemistry and Earth Sciences, College of Arts and Sciences, Qatar University, Doha 2713, Qatar; ia1703713@student.qu.edu.qa (I.N.); s.hersi@qu.edu.qa (S.S.H.); aelshafie@qu.edu.qa (A.S.E.-S.)

**Keywords:** clofazimine, green adsorbent, wastewater, olive stones biochar, magnetized biochar, box–behnken design

## Abstract

Olive stone biochars (OSBC), both pristine and following magnetization (MAG–OSBC), were utilized as eco-friendly and cost-effective sorbents for the antituberculosis, clofazimine (CLOF). Morphologies, textures, surface functionalities, and thermal stabilities of both adsorbents were explored using SEM, EDX, TEM, BET, FT-IR, Raman, XRD and TGA analyses. SEM analysis showed meso- and macroporous surfaces. BET data showed that the MAG–OSBC possesses a larger surface area (33.82 m^2^/g) and pore volume. Batch adsorption studies were conducted following the experimental scenario of Box–Behnken (BB) design. The adsorption efficiency of both adsorbents was evaluated in terms of the % removal (%R) and the sorption capacity (*q_e_*, mg/g). Dependent variables (%R and *q_e_*) were maximized as a function of four factors: pH, sorbent dose (AD), the concentration of CLOF ([CLOF]), and contact time (CT). A %R of 98.10% and 98.61% could be obtained using OSBC and MAG–OSBC, respectively. Equilibrium studies indicated that both Langmuir and Freundlich models were perfectly fit for adsorption of CLOF. Maximum adsorption capacity (*q_max_*) of 174.03 mg/g was obtained using MAG–OSBC. Adsorption kinetics could be best illustrated using the pseudo-second-order (PSO) model. The adsorption–desorption studies showed that both adsorbents could be restored with the adsorption efficiency being conserved up to 92% after the sixth cycles.

## 1. Introduction

Pharmaceutically active compounds (PhACs) and pharmaceutical and personal care products (PPCPs) represent a significant category of emergent pollutants that have enticed the attention of the scientific community, health authorities, and the public. With the increased health awareness, the daily use of PhACs and PPCPs is becoming indispensable. The 2017 report issued by the Organization for Economic Cooperation and Development has mentioned that the expenditure on wholesale pharmaceuticals per capita averaged 564 $/person in the USA and that 75% of this figure was for prescription drugs. Possessing disparate chemical structures, most such compounds cannot be removed effectively by conventional wastewater treatment technologies. Considering their hazardous impacts not only on human and animal health but on environmental safety as well, the existence of PhACs and PPCPs (in water, food, etc.) represents a challenge [1,2,3].

**Scheme 1:**
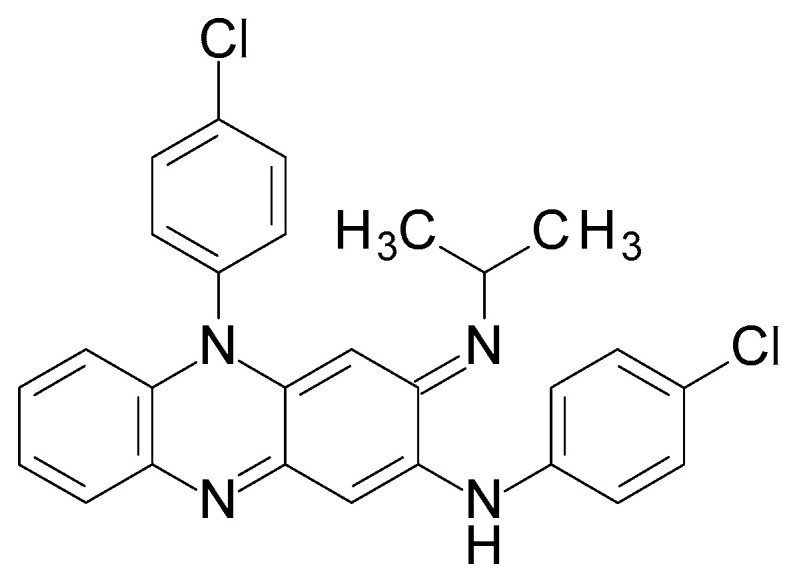
Chemical structure of clofazimine (CLOF). Chemical Formula: C_27_H_22_Cl_2_N_4_ (473.4 g/mol); IUPAC Name: (N,5-bis(4-chlorophenyl)-3-propan-2-yliminophenazin-2-amine; pK_a1_:9.29, pK_a2_:2.31 [10].

Clofazimine (CLOF), a member of the riminophenazine family, is an antimicrobial drug that is commonly used to treat leprosy and drug-resistant tuberculosis (TB). As per the WHO (World Health Organization), CLOF is listed as one of the essential medicines (safest and most effective list of medications) [4,5]. Recent investigations following the COVID–19 pandemic showed that CLOF and thanks to its remarkable anti-inflammatory and immunomodulatory effects, could be used as a therapy in severe COVID-19 cases [6,7,8,9]. Scheme 1 shows the relevant physicochemical data of CLOF [10]. Before the current pandemic, TB was classified as one of the leading causes of death in the world. In 2013, 1.5 million TB-associated deaths were reported. One of the major reasons behind such a crisis is the emergence of drug-resistant TB [11,12]. By and large, these numbers are alarming. On one hand, the existence of these antimicrobials in wastewater may lead to the appearance of antibiotic-resistant strains of TB [13,14]. On the other hand, a literature survey shows little effort being reported to remove the anti-TB drugs from wastewater. Efforts were mainly focused on using advanced oxidation processes (AOPs), electro–Fenton and photoelectro–Fenton technologies [15,16].

Possessing contemporary surface properties, carbon-based materials (CBMs) and especially those obtained from a natural precursor (recycled agro-wastes) are among the most widely investigated adsorbents [2]. Applications included the removal of organics, e.g., PhACs, pesticides, dyes, etc. [13,14,15,16,17,18,19,20,21,22], as well as inorganics [23,24]. By and large, biochars obtained from agro-wastes possess high surface area, unique pore size and pore distribution, in addition to being cost-effective. Moreover, with their lignocellulosic origin, the existence of various functional groups on their surface and the potential for further functionalization facilitate a strong interaction with the target pollutant.

Yet, the difficulty of separating the powdered biochar from the environmental medium hinders the application of these adsorbents on a wide scale and may lead to secondary pollution. Magnetization of carbonaceous wastes helps to overcome this problem. The introduction of the magnetite (or other transition metals and their oxides) creates a separable adsorbent. Moreover, the presence of magnetic nanoparticles with their small particle size, high surface area: volume ratio, fast adsorption kinetics, and potential for recovery and regeneration help to create a unique adsorbent [14,25,26,27].

Olive oil production is a well-established industry. As per the International Olive Council, Spain, as a major producer of olive oil, produces around 625,600 tons/year [28]. The Food and Agriculture Organization (FAO) data report of 2019 shows 21 million tons annual production of olives worldwide [29]. Yet, this industry and with the production of various byproducts are facing serious sustainability problems. While only 20% of the original olive mass is converted into olive oil, the fate of ~50% is olive mill wastewater, and ~30% will be a solid waste (pulp and pit) [30]. These byproducts represent a burden on the environment if not properly managed. Though an escalating interest in the use of the biochar of olive pits can be glimpsed in the literature, yet their use as a cost-effective adsorbent, especially for the removal of the PhACs, remains underestimated. Table 1 shows a summary of the approaches that have used biochar of olive stones for the treatment of pharmaceutical wastewater as well as other organics [31,32,33,34,35,36,37]. As shown in Table 1, most of the reported approaches, if not all, involve the removal of PhACs, dyes and pesticides using pristine or chemically modified biochar of olive stones and follow a univariate-based approach for investigating the process variables. This approach is not only consuming time and resources but also does not preserve the method’s greenness.

The current investigation targets developing an economic adsorbent and an eco-structured adsorption approach. The goal will be set to preserve the adsorbent’s cost-effectiveness and the method of greenness. Consequently, the variables that influence the adsorption of CLOF will be optimized. Box–Behnken (BB) design will be, therefore, utilized to tune the process variables; pH, the dose of the biochar (AD), contact time (CT) and concentration of CLOF [38,39]. Two adsorbents will be prepared: pristine olive stone biochar (OSBC) and magnetic biochar (MAG–OSBC). The impact of the aforementioned variables on the performance of both adsorbents will be assessed. The novelty of the current endeavor, therefore, stems from being the first report on using waste-derived materials via an eco-structured approach for the removal of CFZ. In the same itinerary, and since the adsorbent recycling and reuse are essential features from the industrial point-of-view, the reusability and regeneration of both adsorbents will be attested.

## 2. Materials and Methods

### 2.1. Materials, Equipment, and Software

Chemicals used in this study including sodium hydroxide, hydrochloric acid, acetic acid, sodium chloride, nitric acid, sulfuric acid, sodium carbonate, ethanol, ferrous ammonium sulfate hexahydrate (Fe(NH_4_)_2_(SO_4_)_2_·6H_2_O), and ammonium iron (III) sulfate dodecahydrate (NH_4_Fe(SO_4_)_2_·12H_2_O) were purchased from Sigma-Aldrich (St. Louis, MO, USA). Clofazimine (CLOF) was purchased from Biosynth^®^ Carbosynth Ltd. (Compton, Berkshire, UK). Olives were purchased from local supermarkets in Doha, Qatar. Stones were dried in an oven (Memmert, GmbH + Co. KG, Schwabach, Germany) and burnt in the Thermolyne^TM^ furnace (Barnstead, Dubuque, IA, USA). Millipore-Q water system was used to obtain the deionized water used throughout this study. To prepare the CLOF stock solution (100 ppm), the drug was dissolved using few drops of acetic acid, and the volume was made up to the mark using deionized water. The pH of water in which the adsorbents were suspended was adjusted using either 0.1 M NaOH or 0.1 M HCl. The pH measurements were made using a Vernier LabQuest pH meter. Concentrations of CLOF before and after adsorption were measured using a UV-vis spectrophotometer (Agilent diode-array, Agilent, Santa Clara, CA, USA) with 10 mm matched quartz cuvettes. A Millex syringe filter (nylon, nonsterile, 0.45 µm) was used to filter solutions.

To determine the functional groups on the surface of the adsorbent, FT-IR spectroscopy (FT-IR, PerkinElmer, Shelton, CT, USA) was utilized. The surface morphology of both adsorbents was examined using a scanning electron microscope (SEM, FEI, Quanta 200, Thermo Scientific, Waltham, MA, USA) equipped with an energy-dispersive X-ray spectrometer (EDX). The latter was used to identify the elemental composition of both adsorbents. Raman spectroscopy was used to study the nature of the carbonaceous material (Thermo Scientific, Waltham, MA, USA). Transmission electron microscope (TEM, FEI, TECNAI G2 TEM, TF20) was used for microstructural characterization of OSBC–MAG. To further study the surface characteristics, pore size, surface area and volume were measured using a Micrometrics ASAP2020 accelerated surface area and porosimetry system. Degassing of samples was conducted, followed by the N_2_ adsorption–desorption study. To calculate the surface area, the isotherms measured at 77 K were used along with applying the Brunauer–Emmett–Teller (BET) equation. To find the pore volume, *t*-plots were used with Barrett–Joyner–Halenda (BJH) equations. The X-ray diffraction pattern (XRD) was explored on an X-ray diffractometer (X’Pert-Pro MPD, PANalytical Co., Almelo, the Netherlands) using Cu Kα X-ray source (λ = 1.540598 Å) operated over a 2 h range of 5–80° (2θ).

Minitab^®^19 software was purchased from Minitab Inc. (State College, PA, USA) and was used to construct and analyze the BB design.

### 2.2. Preparation of Olive Stone Biochar (OSBC)

Olive stones were removed from olives and were washed five times with tap water first, followed by the other five times using distilled water. After washing, the clean stones were placed in the oven at 80 °C for three consecutive days. Following this treatment, portions of the olive stones were placed in crucibles and left in the oven at 500 °C for 1 h. The stones were crushed using an agate mortar and a pestle, finely divided, and then sieved using an 0.125 mm sieve, and the obtained powder was placed in a sealed bottle and kept for further use.

### 2.3. Preparation of Magnetic Olive Stone Biochar (MAG–OSBC)

For magnetite (Fe_3_O_4_) preparation, the co-precipitation method was utilized [14,40] with minor modifications. A 200 mL of 0.1 M Fe^3+^ solution was mixed with a 100 mL of 0.1 M Fe^2+^ solution. An amount of 10.0 g of the sieved OSBC was suspended in the Fe^3+^/Fe^2+^ mixture. The suspension was stirred for 2 h, and 1 M NaOH solution was added dropwise to pH ~12. The mixture was left at room temperature for 30 min and later was washed with distilled water and then ethanol (five times each), and an external magnet was used to separate the MAG–OSBC. Each step was accompanied by separation using the magnet followed by decantation. Following these washing cycles, the product was dried in the oven at 80 °C overnight. The dried product was kept in sealed bottles for further use.

### 2.4. Determination of the Point-of-Zero-Charge (pH_PZC_)

For the determination of pH_PZC_, equal amounts (1.0 g) of either OSBC or MAG–OSBC were added to a set of seven flasks, each containing 50 mL of 0.01 M NaCl. The pH in each flask was adjusted to values between 3.0 to 9.0 ± 0.2 using either 0.1 M HCl or 0.1 M NaOH. Samples were left to equilibrate for 48 h in the automatic shaker at 150 rpm before measuring the final pH, and an intersection point of the curve (pH_final_ versus pH_initial_) is the pH_PZC_ value [17].

### 2.5. Batch Adsorption Experiments (Response Surface Design)

In the current investigation, Box–Behnken (BB) design was used to optimize the adsorption process variables for each of the two tested adsorbents. Four factors were tested: pH, [CLOF], AD, and CT, Table 2 (lower bound is −1, while the upper bound is +1). The objective was to maximize the percentage removal (%R) and the adsorption capacity (*q_e_*_,_ mg/g). These two parameters were used to assess the adsorptive power of the two adsorbents and were calculated using Equations (1) and (2). The design output involved 27 runs comprising 3 central points (Ct Pt). The design was conducted over 3 blocks, Table 3.

(1) $$\left(\%R\right)=\;\frac{C_{0}-C_{e}}{C_{0}}\;\times 100\%$$

(2) $$\left(q_{e}\right)=\;\frac{C_{0}-C_{e}}{W}\;V$$

where *C*_0_ (ppm) symbolizes the initial concentration of [CLOF] solution, *C_e_* is the concentration of the [CLOF] solution at equilibrium, V is the volume of the solution (L), and W is the weight of the adsorbent used (g).

Equation (3) will be fitted to the data shown in Table 3, and the output will be the regression models that relate the measured response(s) to the input variables.

(3) $$Y=b_{1}+\overset{n}{\underset{i=1}{\sum }}\;b_{i}\cdot X_{i}+\overset{n}{\underset{i=1}{\sum }}\;b_{ii}\cdot X_{i}{}^{2}+\overset{n-1}{\underset{i=1}{\sum }}\cdot \overset{n}{\underset{j=i+1}{\sum }}b_{ij}X_{j}\cdot X_{i}+e\;\;$$

where *Y* is the measured response(s), %R and *q_e_* (mg/g), *e* is the error, and *X_i_*, *X_j_* are the input variables. The coefficients *b_i_*, *b_ii_*,*…b_ij_* will be determined from the regression equations.

### 2.6. Equilibrium and Kinetic Studies

For equilibrium studies, a stock solution of 500 ppm CLOF was prepared. Further dilutions (5–400 ppm) were prepared in deionized water, and the pH was tuned to pH 3.00 ± 0.20 using 0.1 M HCl and 0.1 M NaOH. An amount of 0.100 ± 0.005 g of either adsorbent was added to 13 mL of the previously prepared solution. The prepared suspensions were placed in the automatic shaker for an equilibrium time of 24 h at 150 rpm. Solutions were then filtered, and the absorbance was measured at 284 nm.

To investigate the adsorption kinetics, 150 mL of CLOF solution (500 ppm, pH 3.00 ± 0.20) was mixed with ~1.0 g of OSBC with shaking. An aliquot of 10 mL was withdrawn over a total time span of 60 min. After each withdrawal, the solution was filtered, and the absorbance of the filtrate was measured at 284 nm. The same procedure for investigating the adsorption kinetics was repeated using MAG–OSBC.

### 2.7. Desorption and Regeneration Studies

To explore the potential of adsorbent reusability, OSBC (2.0 g) was first equilibrated with 260 mL of 25 ppm CLOF solution over a period of 2 h at room temperature. The mixture was then filtered. The adsorbent was washed with distilled water to remove any non-adsorbed traces of the CLOF and then dried in the oven at 70 °C for 48 h. The previous procedures were repeated with MAG–OSBC using the same conditions. Eluents used in the current study were 0.1 M of HCl, H_2_SO_4_, HNO_3_, Na_2_CO_3_, ethanol as well as deionized water. The desorption experiment was performed by mixing 0.1 g of the CLOF-loaded adsorbent with 10 mL of the eluent. Samples were kept in the automatic shaker for 30 min at 150 rpm. The mixture was filtered, and the absorbance of the filtrate was measured at 284 nm. Each of the desorption experiments was repeated three times, and the average values of the desorbed amount were plotted. Error bars were used to express the standard deviation between the replicate measurements.

Recovery studies were carried using ethanol. An amount of 0.2 g of OSBC was equilibrated with 25 mL of 20 ppm CLOF solution (pH 3.0 ± 0.2) for 1 h at room temperature. The obtained mixture was then filtered, and the absorbance of the filtrate was measured at 284 nm. The loaded adsorbent was eluted using ethanol, and 0.1 M H_2_SO_4_ for OSBC and MAG–OSBC, respectively, and samples were then left in the oven at 70 °C for 1 h, then used for another adsorption cycle. This process was renewed six times, and in each cycle, the removal efficiency (%R) was determined.

### 2.8. Economics and Financial Assessment

To assess the economic effects of the process of biochar production from agro-wastes, it is important to consider the cost of all materials as well as the energy consumption. Compared to the commercial adsorbents, agro-wastes (olive stones in our case) are of no cost. Moreover, upcycling waste material into a value-added product serves to relieve the burden on the environment that could be encountered if wastes were not properly recycled and reused. The estimated energy consumption per kg of activated carbon (OSBC) is 175.65 KWh/kg for an electricity tariff of 0.087 $/KWh (Qatar, 2021). This included energy consumption by the oven and the furnace. The total price per kg is 15.28 $ compared to an average price of 124 $/0.5 kg as per the Sigma-Aldrich website (shipping fees to Qatar are not included). Reagents used for the preparation of 12 g MAG–OSBC was: NaOH, iron (III) and iron (II), with a cost of 0.024 $, 0.88 $, and 0.43 $, respectively. The overall cost per kg of MAG–OSBC is 126.45 $ [29]. Though the cost of the magnetic biochar was higher compared to OSBC, it is important to consider the easiness of separation using an external magnet. Moreover, there is no reference cost for the commercial magnetic biochar, but just thinking about the price of the pristine commercial biochar as a starting material, we can see that the current approach is profitable.

## 3. Results and Discussion

### 3.1. Adsorbent Characterization and Surface Chemistry

#### 3.1.1. Thermogravimetric Analysis (TGA)

**Figure 1:**
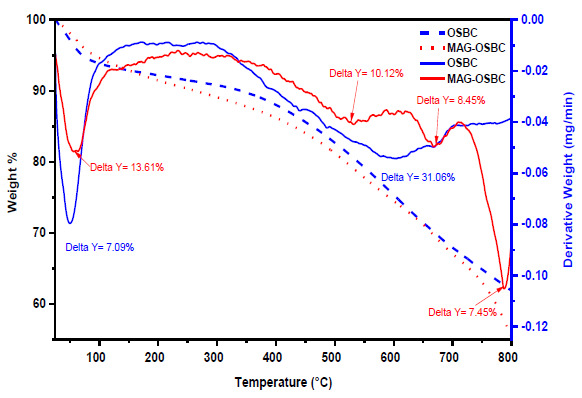
TGA/*d*TA analysis of olive stone biochars (OSBC) and magnetic olive stone biochar (MAG–OSBC).

The thermal stability of both adsorbents was studied using the TGA, Figure 1. The obtained data show that both adsorbents are thermally stable in the range of 100–450 °C. The weight loss between 50 and 100 °C was 7.09% and 13.61% for OSBC and MAG–OSBC, respectively, and could be attributed to the vaporization of free water. A considerable loss, 31.06% and 26.02%, were observed between 550 and 800 °C; for OSBC and MAG–OSBC, respectively, which could be due to the loss of organic matter and the carbonization of the polymeric material. The thermal stability of MAG–OSBC after 550 °C was higher compared to the OSBC due to the presence of magnetite on the surface.

#### 3.1.2. FT-IR Analysis and Point-of-Zero-Charge (pH_PZC_)

**Figure 2:**
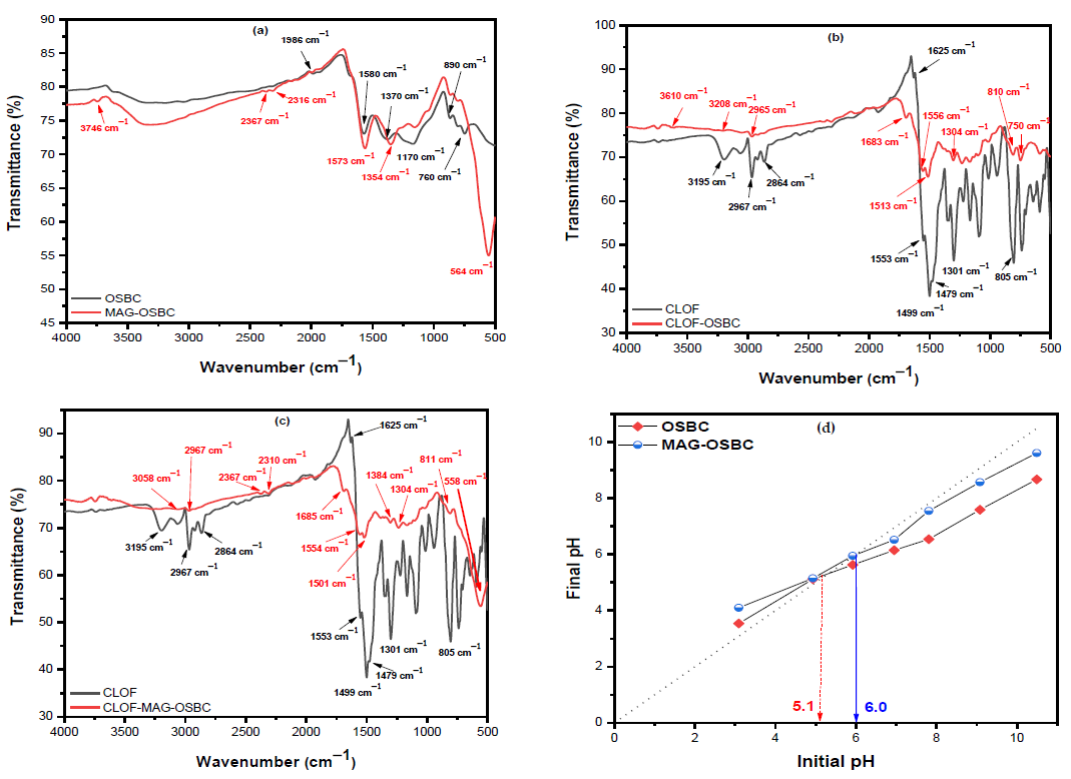
FT-IR spectra of OSBC and MAG–OSBC (**a**) before adsorption, (**b**) free CLOF and OSBC after adsorption, (**c**) free CLOF and MAG–OSBC after adsorption, (**d**) point-of-zero-charge (pH_PZC_) for both adsorbents.

The functional groups on the surface of the prepared adsorbents before and following adsorption of CLOF, as well as for the free CLOF, were determined using FT-IR. Figure 2a shows the IR spectrum of both OSBC and MAG–OSBC before the adsorption. The obtained data show that both adsorbents possess almost similar spectra except for the sharp peak at 564 cm^−1^ in the spectrum of the MAG–OSBC. The presence of this peak is most likely because of the Fe–O bond vibration [41,42,43]. The band at 1580 cm^−1^ in the spectrum of OSBC could be assigned to the aromatic skeletal vibration in lignin. The two absorption bands at 1370 cm^−1^ and 1170 cm^−1^ are related to the C–H deformation and C–O–C vibration, respectively. The absorption band at 890 cm^−1^ corresponds to the C–H deformation in cellulose, and the band at 760 cm^−1^ is attributed to the aryl C–H or the aryl C–O groups [44].

The spectrum of free CLOF is presented in Figure 2b. The spectrum shows strong characteristic absorption bands of the N–H bending frequency at 1550–1620 cm^−1^ and an absorption band at 1625 cm^−1^ corresponding to the C=N stretching vibration [45,46]. Following the adsorption, the spectra of both OSBC and MAG–OSBC show the presence of the CLOF characteristic bands with a different intensity or shifted, implying the adsorption of CLOF onto OSBC and MAG–OSBC, Figure 2c.

Investigation of the pH_PZC_ of both adsorbents showed that OSBC has a pH_PZC_ of 5.1 compared to 6.0 in the case of MAG–OSBC, Figure 2d. These values are comparable to the previously reported values for OSBC [47,48]. Therefore, at a pH value of 3.0 ± 0.2 (lower bound), both adsorbents will have a positively charged surface compared to pH of 9.0 ± 0.2 (upper bound), where both will have a negatively charged surface. On the other hand, CLOF is of ampholytic nature with two pK_a_ values, 2.31 and 9.29, Scheme 1 [10]. Therefore, CLOF will be in zwitterion form in the range of 2.31 < pH < 9.29, and the occurrence of electrostatic interaction between CLOF and either adsorbent within the investigated pH range may not be the best explanation for the adsorption mechanism.

#### 3.1.3. Raman Spectroscopy

**Figure 3:**
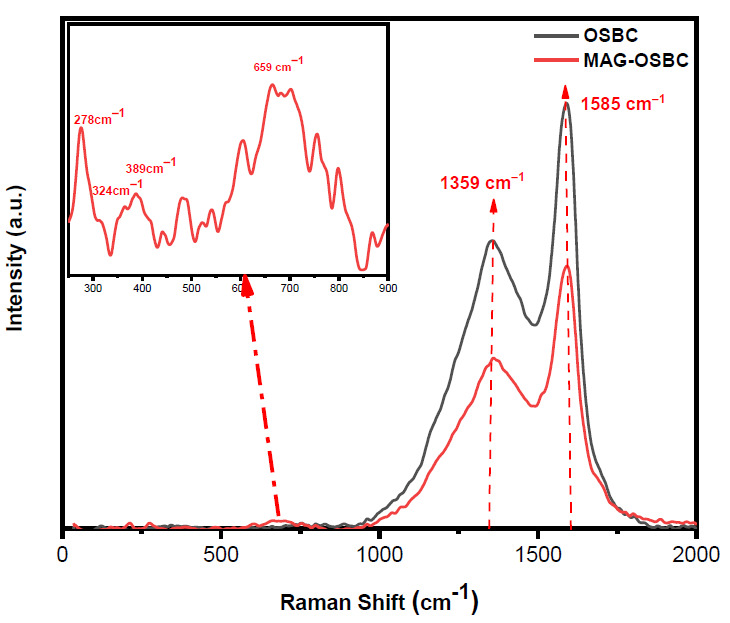
Raman spectra of OSBC and MAG–OSBC**.**

Raman spectra of the two adsorbents are shown in Figure 3. Two distinctive bands usually associated with carbonaceous materials could be observed: at 1359 cm^−1^ (D–band) and 1585 cm^−1^ (G–band). The D–band reflects the carbon lattice characteristics, such as the defects and the sizes, while the G–band reflects the C–C stretching for the *sp^2^* system. The intensity ratio of the two bands, I_D_: I_G_ in the case of OSBC, was 0.680, compared to 0.565 in the case of MAG–OSBC. This finding reveals the presence of defects on the surface of the OSBC, and these defects have decreased following the loading with the magnetic nanoparticles, where the latter serves to cover some of these defects. On the other hand, the spectrum of MAG–OSBC reveals two weak broad peaks centered at 324 and 659 cm^−1^, which are associated with Fe–O bond in magnetite [49,50,51]. The obtained data confirm the formation of the biochar and the presence of magnetite in the impregnated sample.

#### 3.1.4. Textural Properties

**Figure 4:**
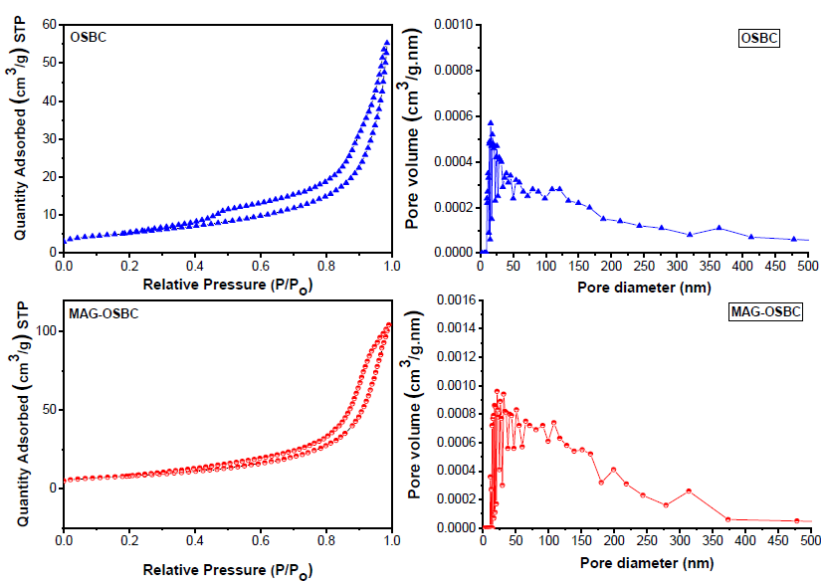
Nitrogen adsorption–desorption isotherms for the prepared adsorbents.

Surface area, pore volume and pore radius as calculated by the Brunauer–Emmett–Teller (BET) method are shown in Table 4. The N_2_ adsorption–desorption isotherms are presented in Figure 4. Obtained data show that the Langmuir surface area has increased from 22.20 m^2^/g in the case of OSBC to 33.82 m^2^/g for MAG–OSBC. This behavior could be attributed to magnetic nanoparticles’ presence on the surface of the OSBC, causing an increase in the surface area, which in turn could improve the removal efficiency towards CLOF. Furthermore, both adsorbents showed the presence of two types of pores: mesopores (2–50 nm) and macropores (>50 nm). The adsorption isotherm was of type IV for both adsorbents, implying monolayer–multilayer adsorption, followed by capillary condensation. The hysteresis loop for both adsorbents was of H3 type. This type is usually found on solids with an extensive pore size distribution, suggesting loose masses of plate-like particles forming slit-like pores [52].

#### 3.1.5. Morphological Characteristics: SEM, EDX, and TEM Analyses

**Figure 5:**
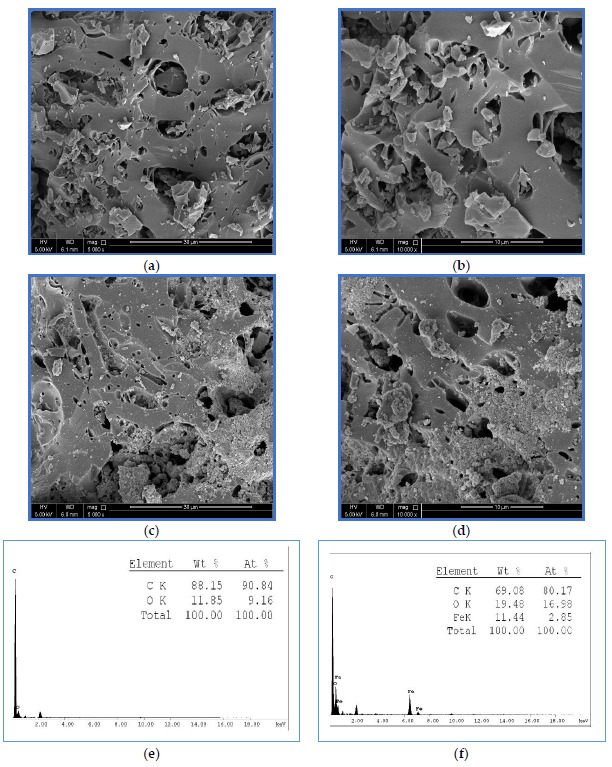
SEM micrographs of (**a**,**b**) OSBC, (**c**,**d**) MAG–OSBC at 5000× and 10,000× magnifications, (**e**,**f**) EDX analyses of OSBC and MAG–OSBC, respectively.

The surface morphology, macroporosity and microscopic features of both adsorbents were visualized using SEM, SEM–EDX and TEM analyses. Figure 5a,b displays the SEM micrographs for OSBC. Shown micrographs prove the presence of different types of pores (meso- and macropores) on the surface of the OSBC, as was confirmed by the BET analysis. For the MAG–OSBC (Figure 5c,d), magnetite nanoparticles appear on the surface, and the size of these particles will be confirmed using the TEM analysis. The SEM findings were further confirmed using the EDX analysis shown in Figure 5e,f. The EDX analysis of the OSBC revealed a high concentration of carbon (88.15%) and oxygen (11.85%), confirming the formation of carbonaceous material following the thermal treatment of the biomass. EDX data for the MAG–OSBC show iron with a concentration of 11.44% and oxygen with a concentration of 19.48%, confirming the formation of iron oxide on the surface of the MAG–OSBC.

**Figure 6:**
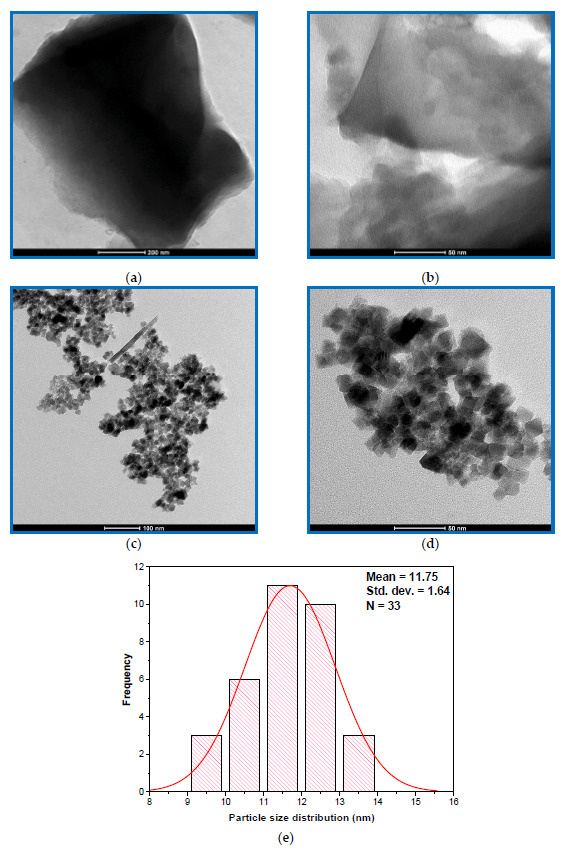
TEM images of the OSBC at two scales (**a**) 200 nm, (**b**) 50 nm and MAG-OSBC at (**c**) 100 nm, (**d**) 50 nm and (**e**) PSD results for the MAG–OSBC.

Microstructural characterization of the as-prepared nanoparticles on the surface of the MAG–OSBC was performed using the TEM analysis, Figure 6. The obtained TEM images agreed with the obtained SEM micrographs. Therefore—and while the surface of the OSBC appears as a clear surface (Figure 6a,b)—that of the MAG–OSBC (Figure 6c,d) looks rough where the magnetic nanoparticles could be easily observed on the surface. The average particle size of these nanoparticles was 11.75 ± 1.64 nm (Figure 6e). The small particle size distribution (PSD) of 1.64 nm confirms the formation of uniform-sized magnetic nanoparticles on the surface of the carbonaceous material.

#### 3.1.6. X-ray Diffraction Analysis (XRD)

**Figure 7:**
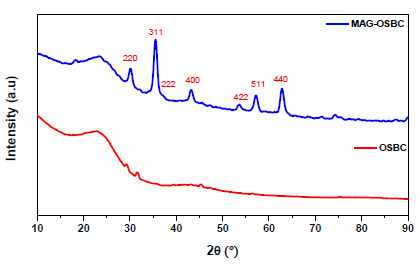
Powder XRD pattern of the OSBC and MAG–OSBC.

X-ray diffraction analysis is an essential analytical technique that could be used to determine the crystalline phase of powdered materials. The samples were analyzed using powder X-ray diffraction analysis to verify the crystalline phase of the as-prepared magnetite nanoparticles. The data shown in Figure 7 represents the XRD diffractogram pattern of both OSBC and MAG–OSBC. The obtained XRD pattern for the OSBC sample shows a broad peak in the range of 2θ 17°–28°, signifying the amorphous state of the OSBC. The same was observed for the MAG–OSBC, confirming the presence of a carbon layer with magnetite nanoparticles [53]. On the other hand, the XRD pattern of the MAG–OSBC shows three intense peaks that could be assigned to cubic Fe_3_O_4_ (ICDD: 98–015–8743) at 2θ 30.15°, 35.59°, and 57.27°. These findings are in good agreement with the similar observation that was previously reported [53,54]. The obtained findings confirm the presence of cubic Fe_3_O_4_ magnetic nanoparticles on the surface of the MAG–OSBC. The XRD analysis can also be used to determine the particle size using the Scherrer Equation (4) [55]. The prepared sample’s crystal size can be calculated from line broadening of the tested sample XRD pattern.

(4) $$D=\frac{K\lambda }{B\;cos\theta }$$

where *λ* is the X-ray wavelength that equals 0.15406 nm, *B* is the corrected width of the major XRD peak of the studied sample at the half-height and at each corresponding angle *θ*, and *K* is a shape factor in this case, equals 0.89. The average particle size *D* (nm) of the prepared magnetite was estimated from XRD line broadening using the Scherrer equation, and it was found to be 22.66 nm, which is close to the obtained data from PSD of the TEM analysis.

### 3.2. Box–Behnken (BB) Design

Batch adsorption experiments were conducted following the BB design matrix shown in Table 2. BB design is a second-order response surface design, which is operated at three levels for each variable. This design is utilized if the number of the predictors is between 3 and 12. One of the advantages of the BB design is that variables are not studied at their extreme levels, meaning that the design does not have runs that measure all variables at the highest points and the lowest points simultaneously [14,29,38,39,56]. Compared to the other RSM designs, e.g., the central composite designs (CCD), BB design entails fewer runs [57].

#### 3.2.1. Investigation of the Statistically Significant Variables

**Figure 8:**
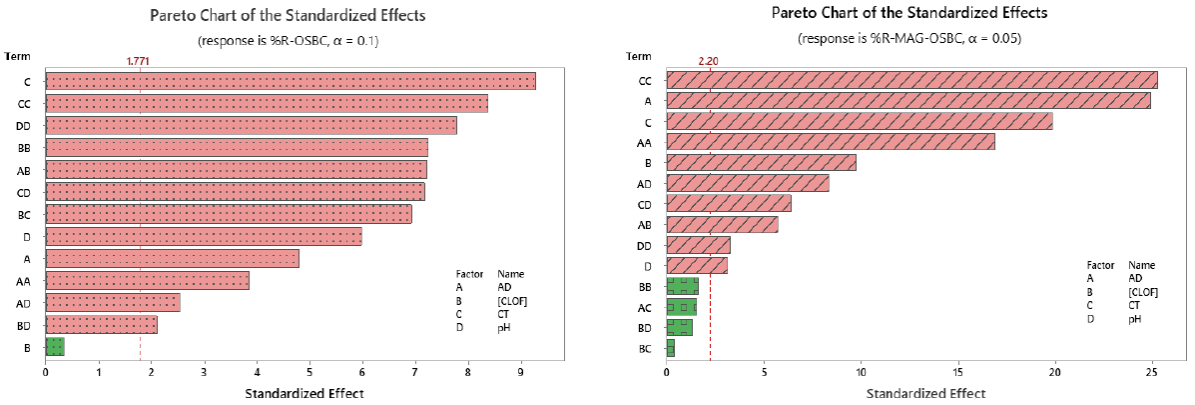
Pareto chart of standardized effects when %R is the optimized response.

To investigate the significance of the studied factors, the Pareto chart of standardized effects was constructed, Figure 8. When %R is the maximized response, the CT (C—linear in case of OSBC and squared in case of MAG–OSBC) was the most statistically significant factor. The magnitude of the impact of pH (D) was much less significant on both adsorbents. With *q_e_*, however, the impact of [CLOF] (B) was the most significant for both adsorbents followed by the AD (A)—Figures are not shown. These findings further confirm the results of FT-IR analysis and the obtained pH_PZC_, where pH has almost no effect on the measured responses, an issue that supports the probability of occurrence of physisorption compared to chemisorption.

#### 3.2.2. Analysis of Variance (ANOVA)

Following the fitting of Equation (3) to the data shown in Table 3, the following polynomial regression models were obtained, Equations (5)–(8). These equations give a clear and comprehensive description of the relationship between dependent and independent variables. Therefore, the overall effect of any variable on the measured response could be simply computed using these equations. The summary of these models is shown in Table 5. The revealed values of the coefficient of determination (R^2^) and R^2^–adjusted (R^2^–adj) are high enough, reflecting the linearity of obtained models. The models’ ability to foretell the response for a new observation is expressed by the value of the R^2^-predicted (R^2^-pred). The high values of the R^2^–pred reflect a reasonable capability of the obtained regression models. The experimental values’ agreement with the predicted ones was expressed by the small values of the relative error (RE), Table 3.

The results for ANOVA testing–Tables are not shown, were in a good match with the findings of the Pareto chart as well as the regression models, where variables with a probability value (*p*-value) of <0.05 (confidence level 95.0) are statistically significant.

%R*_(OSBC)_* = 96.3 + 1.079 AD + 2.565 [CLOF] − 0.034 CT − 34.87 pH − 0.00423 AD × AD − 0.02581 [CLOF] × [CLOF] − 0.01168 CT × CT + 1.926 pH × pH − 0.01649 AD × [CLOF] + 0.0484 AD × pH + 0.01781 [CLOF] × CT − 0.0722 [CLOF] × pH + 0.1538 CT × pH (5)

*q_e(OSBC)_* = 4.44 − 0.0515 AD + 0.4048 [CLOF] + 0.0525 CT − 1.804 pH + 0.000393 AD × AD − 0.000883 [CLOF] × [CLOF] − 0.000636 CT × CT + 0.0794 pH × pH − 0.002491 AD × [CLOF] − 0.000592 AD × CT + 0.00725 AD × pH + 0.000819 [CLOF] × CT − 0.01020 [CLOF] × pH + 0.00743 CT × pH (6)

√%R_(*MAG–OSBC*)_ = −2.513 + 0.14058 AD + 0.0258 [CLOF] + 0.13486 CT + 0.7086 pH − 0.000546 AD × AD − 0.000158 [CLOF] × [CLOF] − 0.000909 CT × CT − 0.02002 pH × pH − 0.000337 AD × [CLOF] − 0.000056 AD × CT − 0.003941 AD × pH + 0.000023 [CLOF] × CT − 0.001130 [CLOF] × pH − 0.003535 CT × pH (7)

ln _(*q_e(MAG–OSBC)_*)_ = −1.407 + 0.00157 AD + 0.08076 [CLOF] + 0.03451 CT + 0.1622 pH − 0.000024 AD × AD − 0.000580 [CLOF] × [CLOF] − 0.000228 CT × CT − 0.00463 pH × pH − 0.000069 AD × [CLOF] − 0.000023 AD × CT − 0.000709 AD × pH + 0.000008 [CLOF] × CT − 0.000472 [CLOF] × pH − 0.000893 CT × pH (8)

#### 3.2.3. Response Optimization

**Figure 9:**
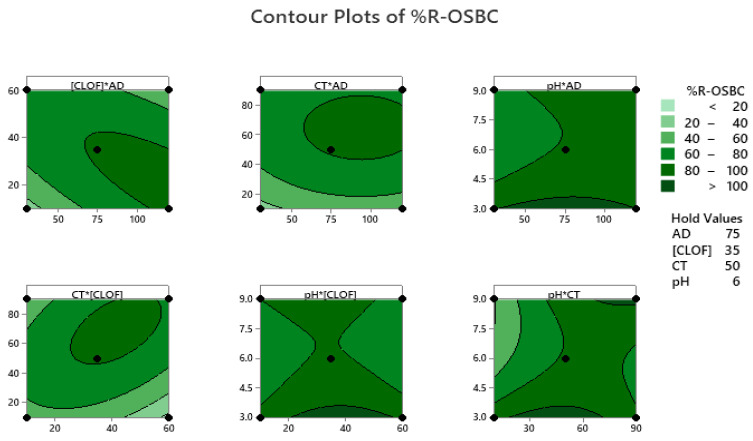
Sample contour plots when %R is the response, using OSBC as an adsorbent.

Contour (2D) and surface (3D) plots were used to ascertain the impact of a certain set of variables on the measured response(s). Sample contour plot when %R is the response being measured and OSBC is the adsorbent shown in Figure 9. The legend on the right of the plots shows the range of %R and the corresponding color. Figure 9—upper left graph shows the impact of the combination of two variables, [CLOF] and the AD, represented on the *y-*and *x-*axis, respectively. As shown, a %R of 80–100% could be achieved using an AD of 60–120 mg and [CLOF] of 0–40 ppm.

Optimization of the individual responses was performed using the response optimizer tool provided by Minitab^®^. Optimum conditions (factorial combinations) that could maximize a response are shown in Table 5. The favorability of any factorial combination was assessed by the value of the desirability function (*d*), where the closer the value of *d* to 1.000, the better the combination. Obtained *d* values are also reported in Table 5 together with the maximum responses obtained using these factorial combinations [58].

### 3.3. Adsorption Isotherms and Kinetic Studies

By and large, the adsorption capability of an adsorbent is dependent on the functionalities existing on the surface of the adsorbent as well as its surface area. Based on the obtained characterization data and the BB design output, different types of interactions could be proposed for the interaction of CLOF and either adsorbent.

#### 3.3.1. Adsorption Isotherms

Adsorption isotherms can be used to determine the degree of accumulation of the adsorbate on the adsorbent’s surface and the type of interaction between the adsorbate and the adsorbent. Four equilibrium isotherms, including Langmuir, Freundlich, Temkin, and Dubinin–Radushkevich (D–R), have been used to investigate the adsorption of CLOF on both adsorbents at a constant temperature [59,60,61,62].

**Figure 10:**
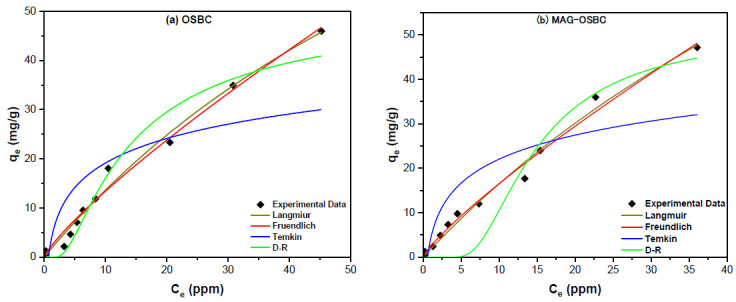
Adsorption isotherms of CLOF onto (**a**) OSBC and (**b**) MAG–OSBC, including Langmuir, Freundlich, Temkin, and Dubinin–Radushkevich (D–R) isotherms.

Langmuir isotherm usually indicates three hypotheses: (I) all adsorption sites on the adsorbent have constant adsorption energy, (II) each adsorbate molecule occupies only one site on the adsorbent and no interaction between the adsorbate molecules, lastly (III) the adsorption is localized. It can be presented by Equation (9) and Figure 10a for the OSBC and Figure 10b for the MAG–OSBC.

(9) $$q_{e}=\frac{q_{m}\;K_{L}\;C_{e}}{1+K_{L}\;C_{e}}$$

where *q_m_* is the maximum adsorption capacity and *K_L_* is the Langmuir equilibrium coefficient. In addition, the Langmuir model can be presented using the following dimensionless format, Equation (10):

(10) $$R_{L}=\frac{1}{1+K_{L}\;C_{0}}$$

where *R_L_* and *C*_0_ (ppm) are the separation factor and initial concentration of CLOF, respectively. Based on the literature, the *R_L_* value can determine the adsorption favorability; thus, if *R_L_* is ˃1, then the adsorption process is considered as unfavorable, while if the *R_L_* = 1, then the adsorption is linear and if the value is between 0 and 1, the adsorption is favorable and can occur spontaneously. However, if the *R_L_* value is zero, then the adsorption is irreversible. The obtained *R_L_* value for OSBC and MAG–OSBC was found to be ˂1, indicating that the adsorption process was spontaneous on both adsorbents and at higher concentrations of CLOF, the process became irreversible with maximum adsorption capacity (*q_max_*) = 137.90 and 174.03 mg/g for OSBC and MAG–OSBC, respectively. This finding shows that the MAG–OSBC as an adsorbent possesses a higher adsorptive capability for CLOF, and this behavior could be related to the higher surface area and pore volume compared to the pristine OSBC. Studies using zinc chloride-activated carbon prepared from olive stone (ACOS), magnetic adsorbent from the olive kernel (MA-OK), and olive stones chemically activated with phosphoric acid showed that Langmuir isotherm could best describe the adsorption of the investigated organic pollutants [33,35,36].

The obtained data show that the performance of the current adsorbents is comparable to the conventional adsorbents. For example, the reported *q_max_* of a commercial activated carbon (Filtrasorb 400) towards diclofenac was 180 mg/g [63]. The performance was even better when compared to the multi- and single-walled carbon nanotubes towards hydrochlorothiazide with *q_max_* of 66.22 mg/g and 45.66 mg/g, respectively [64].

The Freundlich isotherm is a purely empirical approach that can be used to describe the energy of the heterogeneous surface, and it is given by Equation (11):

(11) $$q_{e}=\;K_{F}C_{e}^{\frac{1}{n}}$$

where *C_e_* is the equilibrium concentration of CLOF (ppm); *q_e_* is the amount of CLOF adsorbed/unit mass (mg·g^−1^), *K_F_* (mole·g^−1^)(L·mole^−1^) and 1/*n* are the Freundlich coefficients that express the adsorbent capacity and change in the intensity of the adsorption, as well as the deviation from linearity, Figure 10a,b for the OSBC and MAG–OSBC, respectively and their values, are listed in Table 6. The obtained data from the Freundlich isotherm showed a good fit with an R^2^ = 0.984 and 0.988 for both OSBC and MAG–OSBC, respectively, which is similar to the R^2^ values obtained for the Langmuir isotherm (R^2^ = 0.986 for OSBC and 0.988 for MAG–OSBC), implying that both Langmuir and Freundlich isotherms can be used to describe the adsorption of CLOF onto both adsorbents. Table 6 shows that the values of the 1/*n* = 0.82, *n* = 1.22 for OSBC and 1/n= 0.83, n = 1.20 for the MAG–OSBC. Accordingly, the adsorption potential (A= nRT) = 4.02 kJ, and any CLOF molecule with a potential energy ˂4.02 kJ can be adsorbed onto the surface of MAG–OSBC, and the adsorption tend to be favorable and irreversible. A study using acid-treated olive stones (ATOS) showed that Freundlich isotherm could be best describe the adsorption of the pesticides onto the surface of the ATOS [31].

Temkin isotherm, Figure 10, could provide an idea about the interaction between the adsorbate and the adsorbent, where the heat of the adsorption of adsorbed molecules in a layer decreases linearly with the adsorbent–adsorbate interactions. According to the data presented in Table 6, the sorption energy is 344.6 J/mol for the OSBC and 317.4 J/mol for the MAG–OSBC. These findings imply the favorable adsorption of CLOF onto both adsorbents and confirm the obtained data from Langmuir and Freundlich isotherms.

Finally, the D–R equilibrium isotherm was studied at room temperature, Figure 10, and Table 6. The obtained data for both adsorbents show that the sorption energy for OSBC is 4.271 kJ/mol and 2.969 kJ/mol for the MAG–OSBC, signifying that the adsorption of CLOF onto both adsorbents is physisorption. Moreover, the maximum adsorption capacity of OSBC is 149.30 mg/g, which is aligned with Langmuir’s maximum adsorption capacity.

#### 3.3.2. Kinetic Studies

**Figure 11:**
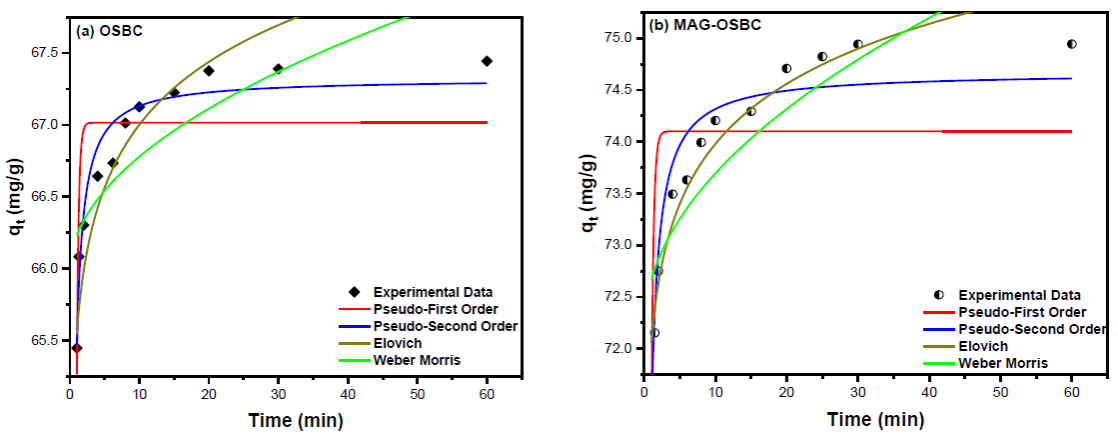
Kinetic models for the adsorption of CLOF onto (**a**) OSBC and (**b**) MAG–OSBC.

Four kinetic models were used to study the adsorption mechanism of CLOF onto both adsorbents, namely pseudo-first-order (PFO), pseudo-second-order (PSO), Elovich, and Weber–Morris (WM) models. The obtained data presented in Figure 11a,b show the relation between *q_t_* (mg/g) versus time (min) for the adsorption of CLOF onto OSBC and MAG–OSBC, respectively. Calculated parameters for the four models are shown in Table 7. The obtained data show that the R^2^ value is higher for the PSO model for both adsorbents (0.9416 for OSBC and 0.9224 for MAG–OSBC). These findings indicate that the rate of the adsorption reaction depends on both drug and adsorbent and that the reaction could be represented as follows, Equation (12):

(12) $$\mathrm{CLOF}+\mathrm{OSBC}\;\mathrm{or}\;\mathrm{MAG}–\mathrm{OSBC}\;\left(\overset{k}{\to }\;\right)\;\left\{\mathrm{CLOF}–\mathrm{OSBC}\right\}\;\mathrm{or}\;\left\{\mathrm{CLOF}–\mathrm{MAG}–\mathrm{OSBC}\right\}$$

In agreement with the literature, and as is shown in Table 1, the adsorption of PhACs and other organics on adsorbents derived from the olive stones followed a PSO kinetic model [31,32,33,34,35,37].

On the other hand, the Elovich model shows significant initial adsorption for both adsorbents, which equals 8.28 × 10^44^ mg·g^−1^·min^−1^ for OSBC and 2.08 × 10^37^ mg·g^−1^.min^−1^ for MAG–OSBC. Finally, the R^2^ value of the Weber–Morris (WM) was too small for both adsorbents compared to the other models (0.6789 and 0.7605 for OSB and MAG–OSBC, respectively); hence this model cannot be used to describe the adsorption of CLOF on the studied adsorbents.

### 3.4. Desorption and Recovery Studies

**Figure 12:**
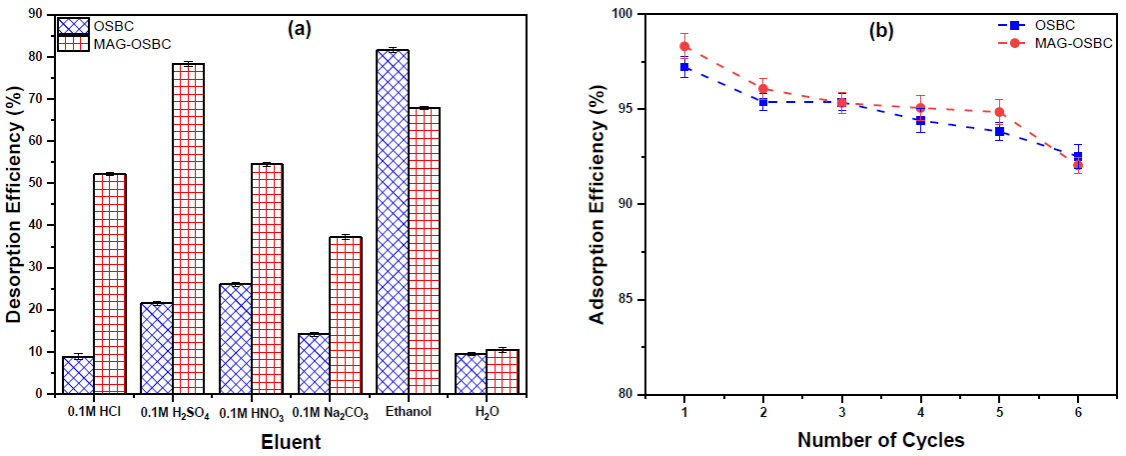
(**a**) Effect of eluent type on the CLOF desorption from OSBC, MAG–OSBC**,** and (**b**) the regeneration performance of both adsorbents for the CLOF removal.

The economic usability for any adsorbent is a significant aspect, and it depends essentially on the adsorbent regeneration. For this purpose, a desorption study was performed using six different eluents, followed by consecutive adsorption–desorption cycles repeated for six cycles [65]. For the desorption study, six eluents were tested for the desorption of CLOF from OSBC and MAG–OSBC adsorbents, including 0.1 M solutions of HCl, H_2_SO_4_, HNO_3_, and Na_2_CO_3_, in addition to ethanol and H_2_O. The data shown in Figure 12a present the relation between the tested eluents versus the desorption efficiency (%). The obtained data show that ethanol is the best eluent for the desorption of CLOF from the OSBC, with a desorption efficiency of 81.56%. On the other hand, the best eluent in the case of MAG–OSBC is 0.1 M H_2_SO_4_ with a desorption efficiency of 78.27%. Accordingly, ethanol and 0.1 M H_2_SO_4_ were further used as the most suitable eluents for desorbing CLOF from OSBC and MAG–OSBC, respectively.

For the adsorbent regeneration study, cyclic adsorption–desorption experiments were conducted, and the resulting data are shown in Figure 12b. The obtained data illustrate that CLOF removal efficiency decreased slightly for both adsorbents, whereas in the case of the OSBC, it decreased from 97.21% (cycle 1) to 92.52% (cycle 6), and for MAG–OSBC, it decreased from 98.31 (cycle 1) to 92.05% (cycle 6). These findings further confirm that both adsorbents are stable and can be regenerated successfully and used for more than six cycles with more than 90% CLOF removal efficiency.

## 4. Conclusions

According to the data presented, efficient and cost-effective adsorbents could be obtained by upcycling olive stones. Two adsorbents were successfully made and utilized in this study, the pristine biochar (OSBC) and the magnetic biochar (MAG–OSBC). Both adsorbents showed a high-efficiency and adsorptive power for clofazimine (CLOF), the widely consumed antituberculosis and antileprotic drug. The removal efficiency hit 98.61% using the MAG–OSBC, which is almost the same as the OSBC (98.10%). TGA analysis showed that the presence of magnetic nanoparticles on the surface had enhanced the thermal stability of MAG–OSBC compared to the OSBC. Characterization of both adsorbents showed that their surfaces possess both meso- and macropores. MAG–OSBC showed a higher surface area (33.82 m^2^/g) and pore volume (0.166 cm^3^/g) than the OSBC. Furthermore, the FT-IR analysis before and after adsorption showed the changes in intensities and shifts in the position of some functional groups confirming the presence of CLOF on the adsorbents’ surfaces. XRD data showed the presence of cubic Fe_3_O_4_ magnetic nanoparticles on the surface of the MAG–OSBC. A multivariate approach was utilized to optimize the dependent responses (%R and *q*_e_), employing Box–Behnken design as a platform. The objective was to achieve maximum removal of CLOF and the highest adsorption capacity of both adsorbents via the lowest possible consumption of chemicals and resources. Design analysis showed that pH plays a negligible role compared to the CT, AD and [CLOF]. Equilibrium studies using nonlinear fittings showed that data fit well to both Langmuir and Freundlich isotherms and that adsorption is favorable with a maximum adsorption capacity (*q_max_*) of 174.03 mg/g in the case of MAG–OSBC compared to 137.90 mg/g using the OSBC. The adsorption was physisorption using both adsorbents. Investigation of adsorption kinetics showed that PSO perfectly fit the adsorption of CLOF onto either adsorbent. The desorption study showed that both adsorbents could be regenerated, with the adsorption efficiency being reserved up to 92% after six cycles. Consumption of materials and energy to produce 1 kg of the biochar showed that the current process is cost-effective and more economical than commercial adsorbents. While the OSBC is easy to prepare cost-effective adsorbent, there is difficulty in removing the powdered adsorbent, an issue that may lead to secondary pollution and could restrict its application on a large scale. The MAG–OSBC, however, and thanks to the magnetism, is separable and, therefore, could have wider scale applications. Yet, its cost-effectiveness and the probable environmental toxicity are issues that should be considered.

## Figures and Tables

**Table 1:** Reported applications of the biochars obtained from olive stones for the removal of pharmaceutically active compounds (PhACs) and other organics.

Adsorbent	Adsorbate	Analytical Approach	Kinetic Model	Isotherm Model	Surface Area (m^2^/g)	*q_max_* (mg/g)	%R	Ref.
Olive stone biochar (OSBC) Magnetic olive stone biochar (MAG–OSBC)	Clofazimine (CLOF)	Batch adsorption, multivariate analysis (Box–Behnken) design	* PSO	Langmuir and Freundlich	22.20 33.82	137.90 174.03	98.10% 98.61%	Current study
Acid treated olive stones (ATOS)	Drin pesticides	Batch adsorption, univariate analysis	* PSO	Freundlich	479	19.54–43.71	˃90%	[31]
Olive stones (OS) and activated carbon olive stones (OSAC)	Methylene blue (MB)	Batch adsorption, univariate analysis	* PSO	Temkin	** NS	OSAC: 16.2 OS: 12.9	OSAC: 94% OS: 70%	[32]
Zinc chloride-activated carbon prepared from olive stone (ACOS)	Remazol red B (RRB)	Batch adsorption,univariate analysis	* PSO	Langmuir	ACOS: 3 ZnCl_2_-activated: 58–790	ACOS: 9.33	ACOS: 75%	[33]
Olive stone biomass (OS)	Alizarin red S (ARS) Methylene blue (MB)	Batch adsorption, univariate analysis	* PSO	Redlich–Peterson	0.16	ARS: 16.10 MB: 13.20	ARS: 85% MB: 75%	[34]
Magnetic adsorbent from the olive kernel (MA-OK)	Amoxicillin (AMX)	Batch adsorption, univariate analysis	* PSO	Langmuir	2188.0	238.1	** NS	[35]
Olive stones chemically activated with phosphoric acid	Paracetamol (PA)	Batch and small-scale column studies, univariate analysis	Vermeulen	Langmuir	1000	100	** NS	[36]
Activated carbon from olive stones (ACOS)	Diclofenac sodium (DCF)	Batch adsorption, univariate analysis	* PSO	BET	83.72	11.0071	** NS	[37]

* PSO: pseudo-second-order, ** NS: not stated.

**Table 2:** Dependent and independent variables together with their levels.

Factors	−1	0	+1
pH (A, pH unit)	3.0	6.0	9.0
Adsorbent dose (AD, B, mg/13 mL)	30.0	75.0	120.0
Initial drug concentration ([CLOF], C, ppm)	10.0	35.0	60.0
Contact time (CT, D, min)	10.0	50.0	90.0
Dependent variables	Percentage removal (%R)		
	Adsorption capacity (*q_e_*, mg/g)		

**Table 3:** Experimental setup using Box–Behnken (BB) design. Observed and predicted values for %R and *q_e_*, as well as the relative error (RE), are shown.

Variables					OSBC						MAG–OSBC					
Trial No	AD	[CLOF]	CT	pH	%R Obs.*	%R Pred.**	RE ***	*q_e_* Obs.*	*q_e_* Pred.**	RE ***	%R Obs.*	%R Pred.**	RE ***	*q_e_* Obs.*	*q_e_* Pred.**	RE ***
01	30 (−)	35 (0)	50 (0)	3 (−)	97.81	98.56	0.01	12.67	11.29	0.12	50.27	48.14	0.04	7.55	7.36	0.03
02	120 (+)	60 (+)	50 (0)	6 (0)	44.37	44.85	0.01	2.88	2.84	0.02	71.18	71.80	0.01	4.99	4.98	0.00
03	30 (−)	10 (−)	50 (0)	6 (0)	27.12	31.65	0.14	1.57	1.97	0.20	56.21	55.44	0.01	2.42	2.35	0.03
04	75 (0)	35 (0)	10 (−)	3 (−)	94.11	93.69	0.00	5.69	5.72	0.00	47.21	46.81	0.01	2.85	2.80	0.02
05	30 (−)	35 (0)	50 (0)	9 (+)	70.79	67.74	0.05	7.85	7.57	0.04	62.67	59.93	0.05	9.02	8.51	0.06
06	120 (+)	35 (0)	90 (+)	6 (0)	81.70	75.15	0.09	1.85	1.42	0.30	77.45	73.51	0.05	2.94	2.78	0.06
07	75 (0)	10 (−)	90 (+)	6 (0)	38.74	43.18	0.10	0.67	−0.12	6.59	86.62	83.65	0.04	1.50	1.44	0.04
08	30 (−)	35 (0)	10 (−)	6 (0)	32.49	33.34	0.03	5.29	5.93	0.11	27.12	26.95	0.01	4.44	4.52	0.02
09	75 (0)	10 (−)	10 (−)	6 (0)	52.30	51.21	0.02	0.90	0.11	7.22	61.61	58.43	0.05	1.07	0.99	0.08
10	120 (+)	35 (0)	10 (−)	6 (0)	42.22	47.57	0.11	2.08	2.14	0.03	54.80	52.27	0.05	2.07	2.03	0.02
11	120 (+)	10 (−)	50 (0)	6 (0)	82.68	82.98	0.00	0.89	1.65	0.46	98.61	100.80	0.02	1.12	1.14	0.02
12	75 (0)	10 (−)	50 (0)	3 (−)	92.12	86.68	0.06	1.59	1.84	0.13	97.00	94.37	0.03	1.67	1.63	0.03
13	75 (0)	60 (+)	50 (0)	3 (−)	97.81	96.47	0.01	10.14	10.16	0.00	84.15	82.38	0.02	9.22	8.91	0.03
14	75 (0)	35 (0)	50 (0)	6 (0)	80.47	81.49	0.01	4.88	4.96	0.02	89.37	91.08	0.02	5.42	5.58	0.03
15	75 (0)	35 (0)	50 (0)	6 (0)	79.71	81.49	0.02	4.83	4.96	0.03	88.00	91.08	0.03	5.27	5.58	0.06
16	75 (0)	10 (−)	50 (0)	9 (+)	82.49	79.75	0.03	1.43	1.61	0.11	91.65	92.60	0.01	1.59	1.67	0.05
17	75 (0)	35 (0)	10 (−)	9 (+)	38.35	39.02	0.02	2.32	2.18	0.07	52.24	55.20	0.05	3.17	3.32	0.04
18	120 (+)	35 (0)	50 (0)	9 (+)	96.74	95.04	0.02	3.66	3.61	0.01	72.18	72.47	0.00	2.96	2.92	0.02
19	120 (+)	35 (0)	50 (0)	3 (−)	97.61	99.72	0.02	3.69	3.41	0.08	95.99	96.78	0.01	3.63	3.70	0.02
20	75 (0)	60 (+)	90 (+)	6 (0)	77.62	77.76	0.00	8.07	8.31	0.03	69.11	70.30	0.02	7.18	7.47	0.04
21	30 (−)	35 (0)	90 (+)	6 (0)	68.74	60.93	0.13	9.32	9.47	0.02	46.46	48.19	0.04	7.02	7.27	0.03
22	75 (0)	60 (+)	50 (0)	9 (+)	66.53	67.9	0.02	6.92	6.88	0.01	73.26	74.75	0.02	7.59	7.93	0.04
23	75 (0)	60 (+)	10 (−)	6 (0)	19.95	14.57	0.37	5.03	5.26	0.04	45.76	46.11	0.01	4.96	4.96	0.00
24	75 (0)	35 (0)	90 (+)	3 (−)	80.02	84.35	0.05	4.85	5.35	0.09	82.13	85.35	0.04	4.98	5.15	0.03
25	30 (−)	60 (+)	50 (0)	6 (0)	63.00	67.72	0.07	14.77	14.36	0.03	53.65	54.72	0.02	13.95	14.01	0.00
26	75 (0)	35 (0)	50 (0)	6 (0)	84.30	81.49	0.03	4.82	4.96	0.03	90.30	91.08	0.01	5.60	5.58	0.00
27	75 (0)	35 (0)	90 (+)	9 (+)	98.10	103.52	0.05	5.05	5.38	0.06	62.70	66.08	0.05	3.80	3.97	0.04

* Obs.: observed readings; ** Pred.: predicted readings; *** RE = ǀ(true value−observed value)ǀ/true value.

**Table 4:** Brunauer–Emmett–Teller (BET) analyses of thermally treated olive stones before and after loading with magnetic nanoparticles, OSBC and MAG–OSBC, respectively.

Parameters	OSBC	MAG–OSBC
Langmuir surface area (m^2^/g)	22.20	33.82
Total pore volume (cm^3^/g)	0.086	0.166
Average pore radius (°A)	86.1	87.7

**Table 5:** Summary of the regression models, Equations (5)–(8).

Optimized Response	R^2^%	R^2^–Adj%	R^2^–Pred%	Optimum conditions
%R *_(OSBC)_*	97.76	95.53	90.42	AD = 100 mg, [CLOF] = 25 ppm, CT = 35 min, pH = 3.0 (*d* = 1.0000, %R = 100%)
*q_e(OSBC)_*	98.81	97.42	93.32	AD = 30 mg, [CLOF] = 60 ppm, CT = 85 min, pH = 3.0 (*d* = 1.0000, *q_e_* = 18.42 mg/g)
%R *_(MAG–OSBC)_*	99.52	98.86	96.65	AD = 110 mg, [CLOF] = 10 ppm, CT = 65 min, pH = 3.0 (*d* = 1.0000, %R = 100%)
*q_e(MAG–OSBC)_*	99.86	99.66	99.05	AD = 30 mg, [CLOF] = 60 ppm, CT = 63 min, pH = 6.0 (*d* = 1.0000, *q_e_* = 14.62 mg/g)

**Table 6:** Nonlinear equations of Langmuir, Freundlich, Temkin and Dubinin–Radushkevich (D–R) isotherms, besides their parameters for the adsorption of CLOF onto both OSBC and MAG–OSBC.

Isotherm	Equations (Nonlinear Forms)	Parameters	Value	
			OSBC	MAG–OSBC
Langmuir	$q_{e}=\frac{q_{m}\;K_{L}\;C_{e}}{1+K_{L}\;C_{e}}$	*q_m_* (mg/g)	137.90	174.03
		*K_L_* (L·mole^−1^)	0.011	0.010
		R^2^	0.9865	0.9879
Freundlich	$q_{e}=K_{F}C_{e}^{\frac{1}{n}}$	1/n	0.82	0.83
		*K_F_* (mole/g) (L/mole)^1/n^	2.00	2.42
		R^2^	0.9842	0.9886
Temkin	$q_{e}=\frac{RT}{b_{T}}\;\ln\left(A_{T}\;C_{e}\right)$	*b_T_* (J/mole)	344.6	317.4
		*A_T_* (L/mole)	1.435	1.685
		R^2^	0.6334	0.7346
D–R	$q_{e}=\;q_{s}$· exp (−β·ε^2^)	β	2.74 × 10^−8^	5.67 × 10^−8^
	$\epsilon =RT\left(1+\frac{1}{C_{e}}\right)$	*E* (kJ/mole)	4.271	2.969
		*q_m_* (mg/g)	149.32	50.99
	E=12β	R^2^	0.9603	0.8792

*q_e_*: amount of adsorbate in the adsorbent at equilibrium, *K_L_*: Langmuir isotherm constant, *q_m_*_:_ maximum monolayer coverage capacities, *K_F_*: Freundlich adsorption constant, *C_e_*: equilibrium concentration, *q_s_*: theoretical isotherm saturation capacity, *A_T_*: Temkin isotherm equilibrium binding constant, *R*: universal gas constant (8.314 J/mol K), *T* is the temperature (K), *b_T_*: Temkin isotherm constant, *β*: is a constant related to the adsorption energy and ϵ: is the adsorption potential.

**Table 7:** Data for the kinetic studies corresponding to the data shown in Figure 11.

Models	Parameter	Value	
		OSBC	MAG–OSBC
Pseudo-first-order (PFO) $\frac{dq_{t}}{dt}$= *k*_1_(*q_e_* − *q_t_*)	*K_1_* (min^−1^)	3.644	3.236
	*q_e_* (mg/g)	67.01	74.10
	R^2^	0.6725	0.5025
Pseudo-second-order (PSO) $\frac{dq_{t}}{dt}$*= k*_2_(*q_e_* − *q_t_*)^2^	*K_2_ (*g·mg^−1^·min^−1^*)*	0.524	0.281
	*q_e_* (mg/g)	67.32	74.67
	R^2^	0.9416	0.9224
Elovich model *q_t_ =* $\frac{1}{\beta }\;\times \ln\left(1+\alpha \beta t\right)$	*α*	8.28 × 10^44^	2.08 × 10^37^
	*Β*	1.585	1.195
	R^2^	0.7761	0.9479
Weber−Morris model (WM) $q_{t}=K_{I}t^{0.5}+C$	*K_I_*	0.255	0.475
	*C*	65.97	72.19
	R^2^	0.6789	0.7605

where *K*_2_ is rate constant (g·mg^−1^·min^−1^) and *q_t_* is adsorbed quantity at time *t*; while *α* and *β* are initial sorption concentration rate (mg·g^−1^·min^−1^), and desorption constant (g/mg), *K_I_* is intraparticle diffusion rate constant (mg·g^−1^·min^−0.5^), and *C* is boundary thickness effect.

## Data Availability

The data presented in this study are available within this article. Further inquiries could be directed to the authors.
